# A rare case of acquired myeloperoxidase deficiency

**DOI:** 10.1002/jha2.89

**Published:** 2021-02-02

**Authors:** Zuzana Melnik, Hugo Cruz, Catarina Lau, Maria Inês Freitas, Maria da Graça Henriques, Margarida Lima

**Affiliations:** ^1^ Laboratory Haematology Service Department of Pathology, Centro Hospitalar Universitário do Porto EPE; ^2^ Microbiology Service Department of Pathology, Centro Hospitalar Universitário do Porto EPE; ^3^ Laboratory of Cytometry, Clinical Haematology Service, Department of Medicine Centro Hospitalar Universitário do Porto EPE; ^4^ Centralized Laboratory (CoreLab), Department of Pathology Centro Hospitalar Universitário do Porto EPE


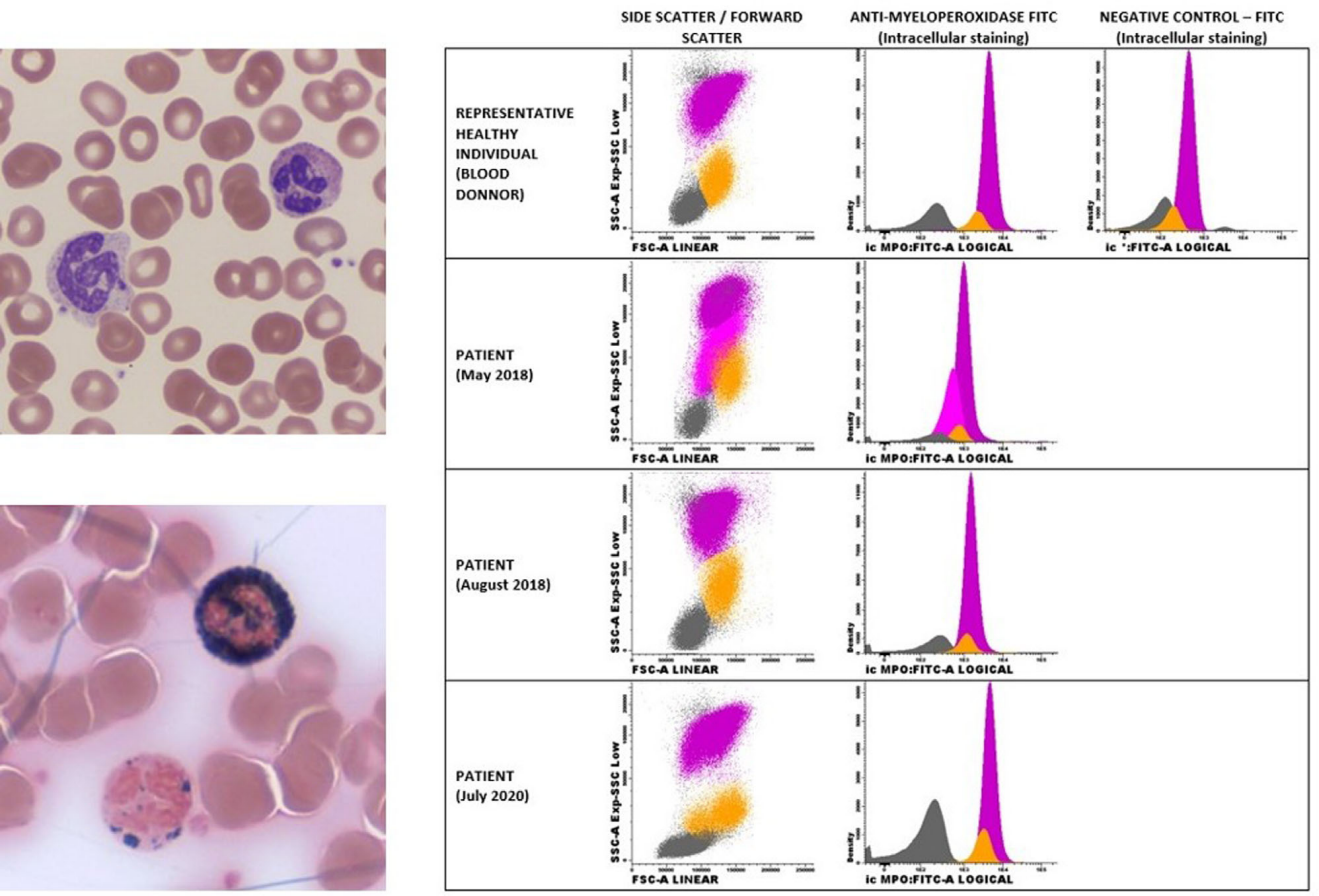


A 49‐year‐old woman with history of kidney transplantation under immunosuppressive therapy and posttransplant diabetes mellitus presented with invasive fungal infection of sphenoid sinus. The leukogram revealed an abnormal scattergram and the peripheral blood film showed a dimorphic neutrophil population, one with normal morphology and other with abnormal nuclear segmentation, decreased chromatin condensation, and cytoplasmic hypogranulation (upper left). Myeloperoxidase (MPO) cytochemical stain revealed different degrees of neutrophil staining intensity, with some of them showing no MPO activity (lower left). Flow cytometry (FCM) immunophenotyping using a fluorescein‐conjugated anti‐MPO monoclonal antibody (right) on the Navios flow cytometer (Beckman Coulter) confirmed that neutrophils and monocytes from the patient had MPO deficiency (11% and 22% of the normal, respectively); in addition, part of the neutrophils (36%) was degranulated, as evaluated by the side scatter (SSC) and this abnormal neutrophil population had lower MPO levels than the remaining neutrophils, with an almost complete MPO deficiency (3% vs 16%, respectively). The patient underwent sphenoid sinus surgery, partially discontinued immunosuppressive therapy, and started antifungal therapy. Laboratory studies repeated 3 months after controlling the fungal infection no longer showed cytological abnormalities, and FCM revealed a normal SSC/FSC distribution and a partial recovery of the MPO deficiency, both in neutrophils and monocytes (30% and 38% of the normal, respectively). A subsequent FCM study performed 2 years later showed a normal SSC/FSC distribution and normal MPO values (110% and 120% of the normal in neutrophils and monocytes, respectively).

MPO deficiency is the most common inherited defect in phagocytes and it is associated with impaired microbial killing.^1^ The acquired form is much less frequent than the hereditary form and, as observed in our patient, it is usually transient and reverses after improvement of the underlying conditions.^1^ Moreover, the acquired form has been described to be partial and to involve only a fraction of the neutrophils. To the best of our knowledge, this is the first case of acquired MPO deficiency documented simultaneously by cytological, cytochemical, and FMC studies. Our studies demonstrate that the acquired MPO deficiency affects all the neutrophils, being more severe in the morphologically abnormal (i.e., degranulated) neutrophil population. In this case, the presence of kidney transplantation under immunosuppressive therapy and diabetes mellitus may have been the triggering factors for the acquired MPO deficiency and the subsequent invasive fungal infection.

REFERENCE1

Pahwa
R
, 
Jialal
I
. Myeloperoxidase Deficiency. [Updated 2020 Jun 25]. In: StatPearls [Internet]. Treasure Island (FL): StatPearls Publishing; 2020 January. Available from: https://www.ncbi.nlm.nih.gov/books/NBK470278/


## Footnotes

1 Myeloperoxidase Deficiency: Roma Pahwa; Pranav Modi; Ishwarlal Jialal [1].
